# P-1432. Assessing Equitable Access to Carbapenemase Production Testing in Los Angeles County Hospitals

**DOI:** 10.1093/ofid/ofae631.1606

**Published:** 2025-01-29

**Authors:** Kelsey OYong, Sandeep Bhaurla, Wendy Knight, Janet A Hindler, Zachary A Rubin

**Affiliations:** Los Angeles County Department of Public Health, Los Angeles, California; Los Angeles County Department of Public Health, Los Angeles, California; Los Angeles County Department of Public Health, Los Angeles, California; Los Angeles County Health Department, Downey, California; Los Angeles County Department of Public Health, Los Angeles, California

## Abstract

**Background:**

Carbapenemase producing organisms (CPO) are the cause of difficult-to-treat infections that result in substantial morbidity and mortality. Due to this and because CPOs can spread quickly between patients, hospital microbiology laboratories should be incentivized to test for carbapenemase production (CP), yet testing costs have stifled uptake. In Los Angeles County (LAC), laboratory professional organizations and the LAC Department of Public Health have broadly promoted CP testing, without emphasis on hospitals that accept patients of highest risk of acquiring a CPO, including patients with a history of long-term care. This analysis assesses CP testing uptake in LAC hospitals and association with patient population and social vulnerability.

**Methods:**

CP testing in hospitals was assessed using the 2022 National Healthcare Safety Network Annual Facility Survey. We assessed patient origin from the Centers for Medicare and Medicaid Services claims and Minimum Data Set and patient characteristics from the California Department of Health Care Access and Information Healthcare Utilization. Frequencies, logistic regression, and Fisher’s exact tests were performed to evaluate the relationship between CP testing and hospital patient and facility-level characteristics.

**Results:**

Of the 79 surveyed hospitals in LAC, 51 (65%) tested for CP. No significant relationship was identified between access to CP testing and the number of patient transfers from high-risk facilities (OR=1.00 , 95% CI [1.00, 1.00]), area poverty (OR=1.01 , 95% CI [0.95, 1.07]), the percent of patients with Medicaid or Medicare (OR=0.94 , 95% CI [0.91, 0.98]), the percent of non-white patients (OR=1.01 , 95% CI (0.99, 1.03]), and hospital ownership (p=0.66).

**Conclusion:**

Access to CP testing was lower among hospitals serving more patients with public insurance and did not differ based on number of high-risk patients. This analysis is limited in that testing capacity was assessed, but data were not available on frequency of testing or implementation of a process to test at-risk individuals. Testing performed at the LAC Public Health Laboratory can supplement testing gaps for facilities of high need. More outreach to hospitals serving highest risk patients to support access to CP testing is needed.

**Disclosures:**

**All Authors**: No reported disclosures

